# Simulated Performance of a Xenohybrid Bone Graft (SmartBone^®^) in the Treatment of Acetabular Prosthetic Reconstruction

**DOI:** 10.3390/jfb10040053

**Published:** 2019-11-22

**Authors:** Carlo Francesco Grottoli, Alberto Cingolani, Fabio Zambon, Riccardo Ferracini, Tomaso Villa, Giuseppe Perale

**Affiliations:** 1 Industrie Biomediche Insubri SA, 6805 Mezzovico-Vira, Switzerland; carlo.grottoli@ibi-sa.com (C.F.G.); alberto.cingolani@ibi-sa.com (A.C.); 2 Politecnico di Milano, Laboratory of Biological Structure Mechanics, Department of Chemistry, Materials and Chemical Engineering “G. Natta”, 20133 Milan, Italy; fabio.zambon@mail.polimi.it (F.Z.); tomaso.villa@polimi.it (T.V.); 3 Department of Surgical Sciences and Integrated Diagnostics, University of Genova, Largo R. Benzi 10, 16132 Genova, Italy; riccardoferraciniweb@gmail.com; 4 IRCCS Ospedale Policlinico San Martino, Largo R. Benzi 10, 16132 Genova, Italy; 5 Ludwig Boltzmann Institute for Experimental and Clinical Traumatology, Donaueschingenstrasse 13, 1200 Vienna, Austria

**Keywords:** total hip arthroplasty, bone substitute, computational model, 3D reconstruction

## Abstract

Total hip arthroplasty (THA) is a surgical procedure for the replacement of hip joints with artificial prostheses. Several approaches are currently employed in the treatment of this kind of defect. Overall, the most common method involves using a quite invasive metallic support (a Burch–Schneider ring). Moreover, valid alternatives and less invasive techniques still need to be supported by novel material development. In this work, we evaluated the performance of SmartBone^®^, a xenohybrid bone graft composed of a bovine bone matrix reinforced with biodegradable polymers and collagen, as an effective support in acetabular prosthesis reconstruction. Specifically, the material’s mechanical properties were experimentally determined (E = ~1.25 GPa, E_f_ = ~0.34 GPa, and Et = ~0.49 GPa) and used for simulation of the hip joint system with a SmartBone^®^ insert. Moreover, a comparison with a similar case treated with a Burch–Schneider ring was also conducted. It was found that it is possible to perform THA revision surgeries without the insertion of an invasive metal support and it can be nicely combined with SmartBone^®^’s osteointegration characteristics. The material can withstand the loads independently (σ_max_ = ~12 MPa) or be supported by a thinner titanium plate in contact with the bone in the worst cases. This way, improved bone regeneration can be achieved.

## 1. Introduction

The number of primary and revisional total hip arthroplasties (THAs) has significantly increased over the last 25 years. Indeed, in North America alone, the number of primary THAs has grown from approximatively 200,000 cases up to almost 600,000, while the number of revisional THAs has increased from 40,000 to 270,000 [1]. The major push, in this respect, is due to the rapidly growing elderly population and the significant quality-of-life improvement ensured by these procedures [2]. THA is the most common technique to treat acetabular and femur fractures, as well as arthritis phenomena, and it is often associated with internal fixation [3]. In general, the need for a total hip replacement occurs when there is damage at the articular cartilage, which can no longer preserve the joint’s anatomical integrity and physiological functionality. Indeed, the cartilage can be damaged by bone fractures; bone necrosis; traumas not involving the subchondral bone but disconnecting the bone to the cartilage layer [4]; cyclic minor traumas due to femur–acetabular impingement [5] or CAM lesions [6]; or inflammatory issues such as primary osteoarthritis, metabolic or septic, rheumatoid, or other immunologically based arthritis [7].

Several surgical approaches are used to treat acetabular fractures. Chakravarty et al. proposed achieving primary stabilization of the acetabular fracture with cannulated screws [8], and Borens et al. proposed adopting cable fixation to obtain fracture reduction [9]. Moreover, another common technique is the open reduction internal fixation, which is performed before THA [10,11,12]. Finally, another clinical practice involves different types of reinforcement rings to fix the acetabular component to the periacetabular bony structures, in place of using the femoral head as an autograft for acetabular reconstruction. This technique avoids inserting an additional component during the revision surgery, even though it can compromise part of the biomechanics of the hip joint system [13].

In general, the implants need to be fixed to ensure long-term stability. This can be achieved using different techniques, such as press-fitting, positioning of bone cement along the femoral stem and backside to the acetabular cup, or the presence of porous coating to encourage tissue growth [5]. Overall, 60% of acetabular components are not cemented when fixed [14,15]. Generally, during the first surgery of THA, bone cement is not used because of its tendency to break during cycling loads, leading to microdebris formation. This can indeed generate inflammation of the surrounding tissue. That is why, in general, residual bone is adapted in order to hold the acetabular component of the prosthesis in place [4].

The correct placement of the acetabular component in the correct anatomical and biomechanically sound position is a key factor to reduce the risk of impingement and dislocation of the implant [16]. Studies by Khatod and colleagues [17,18] showed that the most relevant causes of failure are instability, aseptic loosening, and periprosthetic fracture. The incidence of these was close to 50%, 14% and 11%, respectively. It is also important to note that no infection causes were detected, but they were present in the case of revision surgery [18]. However, the revision approach is not only influenced by pathologies, but it is also connected to the dimension of the damage on the retroacetabular zone [19]. The limitation of these failings can be tackled with different approaches, such as impaction bone grafting, structural allografts, oversized hemispheric cups, and acetabular reconstruction cages [20]. Finally, a different technique adopted by Uchiyama et al. involving a bone allografting technique associated with the use of metallic plates appeared to be better both for new revision surgery and young patients [21].

The long-term performance of these implants is significantly important for avoiding early revision surgeries. The hip prosthesis has to re-establish the correct functionality of the hip–pelvis complex [1]. To restore this function in the best possible way, the hip prosthesis is divided into different components. Each of them provides different attributes and uniquely contributes to the functional requirements of the total hip replacement. These characteristics are related to wear resistance of the acetabular liner and femoral head, articulation, and fatigue resistance of the stem.

This is also possible because of the development of novel materials, which can provide mechanical properties, microstructures (i.e., porosity [22]), and a composition meeting the functional requirements for hip replacement [23,24]. Nevertheless, in general, the appropriate control over these material characteristics as a whole is limited, and complex strategies to control their features need to be developed [25,26,27,28,29]. As a matter of fact, ceramic, metallic, and polymeric materials are nowadays combined to optimize the performance of the implant itself [30,31,32].

Among resorbable materials, SmartBone^®^ (Industrie Biomediche Insubri SA - I.B.I., Mezzovico-Vira, Switzerland) is a commercial xenohybrid bone graft that has been available on the market since 2012 and has the upgraded physical properties of decellularized and deproteinized trabecular bovine bone via polymer and gelatin deposition [33]. Specifically, thanks to the appropriate combination of these components, its mechanical properties are improved and homogenized and it can easily absorb and retain blood once in situ, thus promoting cell colonization and proliferation [34,35]. This is a crucial aspect for the performance of bone grafts, as it ensures appropriate integration within the patient’s tissues [36,37]. As a matter of fact, previous studies [38,39,40] have already highlighted the ability of the material to be osteoconductive and resorbable, supporting the formation of new bone via remodeling only a few months after grafting [39]. Initially applied successfully to over tens of thousands of patients in dental and maxillofacial clinical applications [39], it has now been positively extended to orthopedic cases, both in standard shapes and with custom-made solutions [41,42,43,44,45]. It has always shown appropriate integration and remodeling, combined with remarkable mechanical resistance over time. Indeed, a 2018 study reported a significant bone volume increase of 40% four months postop [42]. A study from 2019 showed an increment of 16 cc of new bone on the treated area of an important craniofacial district [46], and a study in traumatology showed evidence of not only bone regeneration but also anatomically selective remodeling [45].

In this work, we decided to evaluate the performance of SmartBone^®^ as an independent support in THA reconstruction in comparison with one of the most common techniques, which involves using a Burch–Schneider ring. The mechanical properties of the material (compression, torsion, and bending) were experimentally determined and used for simulation. The results showed that SmartBone^®^ can be effectively employed as a structural material in acetabular reconstruction using a more conservative surgery approach and reducing the invasiveness of the most common prosthesis thanks to an easier fixation system. Considering the osteoconductive properties of the graft, further development into real cases seems to be fully possible, opening the way to a novel perspective in prosthesis treatment with completely resorbable biomaterials.

## 2. Results

### 2.1. Mechanical Characteristics of SmartBone^®^

The results of the mechanical characterization of the material under torsion, bending, and compression are summarized in Table 1 and discussed, point by point, below. Details and results for individual samples are reported in the Supporting Information (SI). Specifically, Table S1 and Figure S1 report compression tests, Table S2 and Figure S2 report bending test and Table S3 and Figure S3 report torsion tests.

*Compression*. The results showed that, on average, the material has a high resistance to compression. As a matter of fact, the average maximum stress observed was 25.8 MPa and the mean elastic modulus was 1.2456 GPa. For the sake of completeness, it is worth mentioning that all tested samples showed a yield stress between 10 and 40 MPa except for sample 3 of lot 272, which showed a higher value (Table S1 in SI). Moreover, the values of the elastic modulus were in the range of 0.9–1.8 GPa for all samples. This is not surprising, as, in general, the mechanical properties of porous materials depend on the porosity itself [47]. As the base material of SmartBone^®^ is trabecular bovine bone, the intrinsic natural variability might be reflected in the characterization [48]. On the other hand, variability among samples regarding the stress/strain ratio was not critical and still allowed determination of the representative average values. Indeed, the homogenization of the mechanical properties is one of the major outcomes of coating the trabecular bovine bone with the polymer and gelatin used for SmartBone^®^ preparation [33].

*Bending*. The results obtained for all samples appeared quite homogeneous and, on average, the maximum load was 23.7 MPa and the Young’s modulus was 0.3406 GPa. Moreover, all individual samples showed a yield stress between 19 and 30 MPa except for sample 2 of lot 276, which showed a lower value (Table S2 in SI). Similar considerations were valid for the elastic modulus: the values were in the range of 0.24–0.42 GPa and showed, once again, that there was also no significant variability among the samples regarding the stress/strain ratio for the case of bending.

*Torsion*. The average values of maximum shear stress and tangential modulus were 25.5 MPa and 0.4906 GPa, respectively. All samples showed a yield stress between 15 and 45 MPa and a tangential modulus in the range of 0.25–0.75 GPa. Thus, nonsignificant variability among samples was also recorded for the case of torsion.

**Figure 1:**
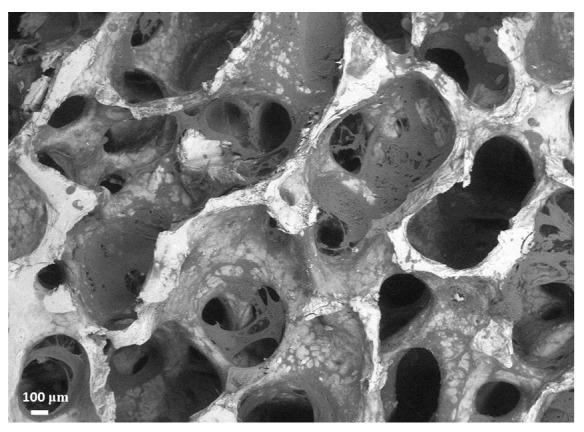
ESEM image of SmartBone^®^ well evidencing its open porous structure and the presence of both the natural mineral structure (white) and the polymeric coating (light gray).

The discussed characteristics are mostly due to the presence of the polymer and gelatin deposited within the mineral porous matrix of trabecular bovine bone, as visible in Figure 1, in which an ESEM (environmental scanning electron microscopy) picture is displayed. As one can see, the deposited polymer is housed within the trabecular framework, reinforcing it but without clog formation and minimally affecting the natural porous structure. In this respect, the polymer’s physical and chemical properties have been selected to rapidly fade away upon implantation, without losing mechanical support and ensuring cell colonization and proliferation over longer periods of time [49].

### 2.2. Pathological Model

**Figure 2:**
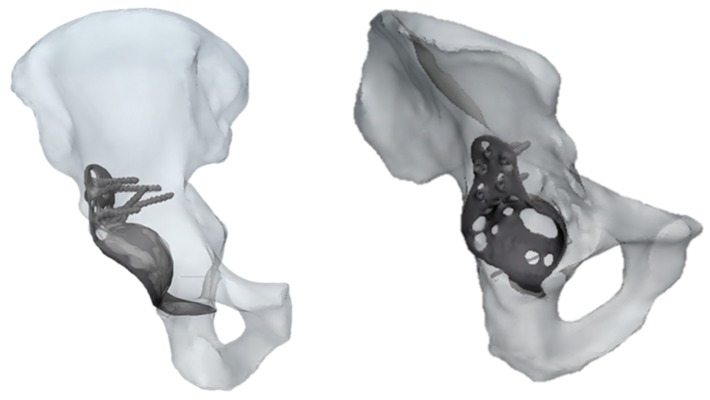
Two different views of pathological CAD model treated with a Burch–Schneider ring.

**Figure 3:**
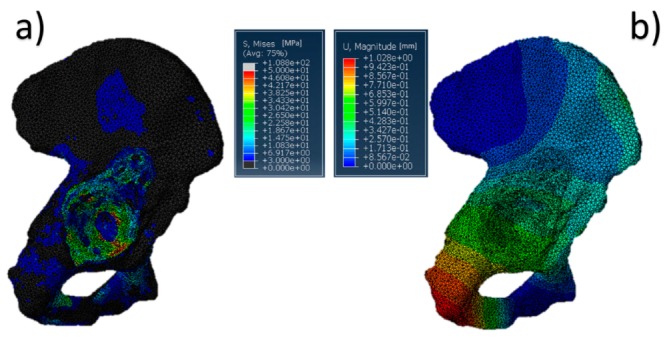
Pelvis pathological model treated with a Burch–Schneider ring: (**a**) von Mises stress distribution (data are expressed in megapascals) and (**b**) displacement distribution (data are expressed in millimeters).

The results referring to the pathological model (Figure 2) treated with a Burch–Schneider ring are shown in Figure 3. All stresses obtained were von Mises ones. The highest values (50 MPa) were found close to the holes on the metallic structure, as visible in Figure 3a. It is important to mention that they are isolated values and do not refer to a wide zone, so, most probably, the overall mean stress is lower than the maximum one found in the plate. Moreover, the plate has different cavities which require screw insertion. Otherwise, unfilled cavities showed higher values of stress.

Finally, a map of the displacement distribution is shown in Figure 3b. Not surprisingly, the value was relatively small (0.5–0.6 mm), also considering the metal nature of the ring itself. Moreover, this can also be dependent on the boundary conditions selected, which fix the model in the distal part of the pubic ramus and the upper part of the ischium.

The insertion of the ring is significantly invasive but functional for a better distribution of the stresses on a pathological and damaged hemipelvis. In fact, from the data obtained, it is possible to notice that the plate absorbs a very wide part of the load applied by the femur in the acetabular zone and provides better stability to the structure. The screws used to anchor the ring to the pelvis might contribute to stress distribution. A minor part of the pressure is transferred to the pelvis but not in the acetabular zone.

### 2.3. Pathological Model Treated with SmartBone^®^ Graft

**Figure 4:**
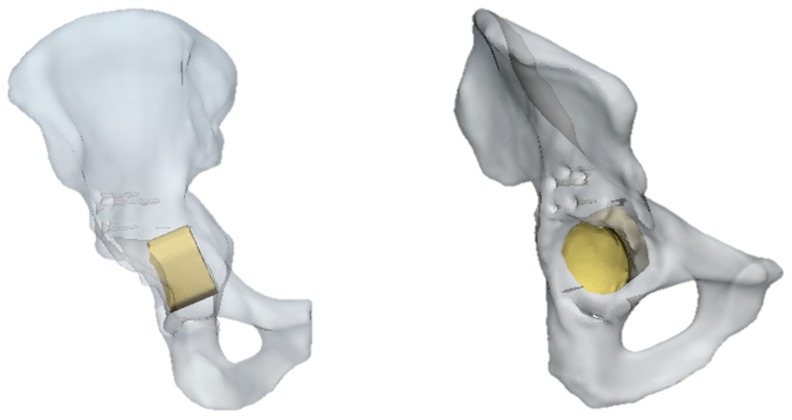
Pelvis pathological model treated with SmartBone^®^.

In this pathological model, the insertion of SmartBone^®^ as a graft was considered, as visible in Figure 4. In general, the simulation had the same general features of the previous one, but in this case, the ring was removed, and the force was applied only on the scaffold of the SmartBone^®^.

**Figure 5:**
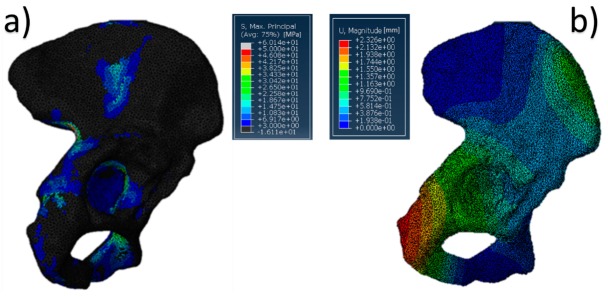
Pelvis pathological model treated with SmartBone^®^: (**a**) von Mises stress distribution (data are expressed in megapascals) and (**b**) displacement distribution (data are expressed in millimeters).

The results of the simulation, in terms of stress distribution and maximum value of displacement, are shown in Figure 5. Overall, the SmartBone^®^ showed significant resistance, as it exhibited a positive behavior under compression forces, confirming the good response obtained during mechanical characterization. In fact, the values reached through applying a distributed load on the graft were under 12 MPa, which was lower than the worst value found during the mechanical tests (Figure 5a). Moreover, the displacement map (Figure 5b) shows that the insert translated about 1–1.5 mm in the direction of the applied hip force, while the deformations on it were negligible.

**Figure 6:**
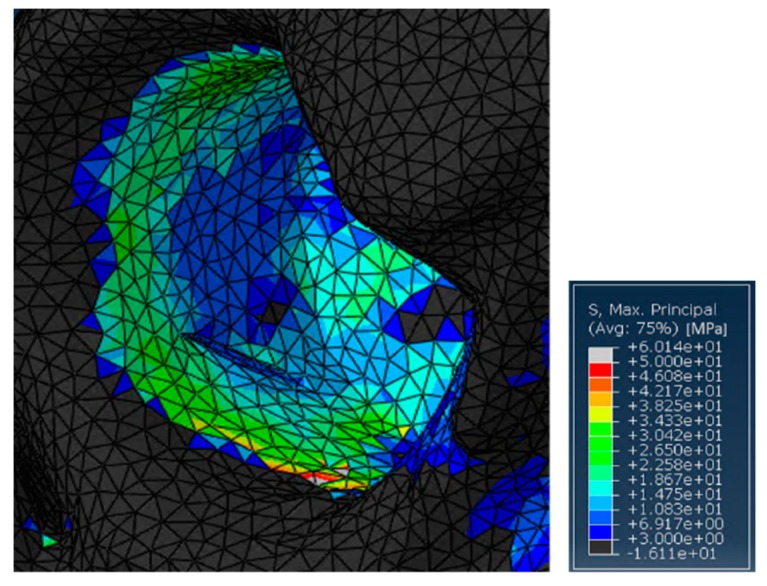
Detail of the stresses on the residual acetabular area in the case treated with SmartBone^®^.

On the other hand, without the ring support, some relatively high stress peaks were found on the retroacetabular area (Figure 6). Indeed, the values reached on the pelvis were around 60 MPa. This is not surprising, as in this case, the insert was directly transferring the load to the residual acetabular area, without the possibility of discharging on a fixation structure. In general, this should not represent a major limitation in SmartBone^®^ usage for this kind of surgery. Nevertheless, it is important to point out that the model might be intrinsically inaccurate because of the impossibility of properly simulating the porosity of the graft itself. Therefore, although this load would not represent an issue in physiological conditions, this might compromise the effectiveness of some revisional surgeries. This would be relevant, for example, in those cases in which the acetabular zone has already been treated by bone chips that do not present significant resistance to compression loads [50].

A possible alternative is, therefore, the combination of a thinner grid interposed between the residual acetabular structure of the pelvis with a SmartBone^®^ insert. This solution can introduce two significant advantages: On the one hand, it has a much smaller weight and dimension with respect to the ring-based techniques, still allowing optimal stability. On the other hand, it has more effective bone regeneration. Indeed, by introducing a SmartBone^®^ graft, cell adhesion is encouraged [43,51], so that integration and bone regrowth is expected to be faster and promoted, in respect to cases treated with the insertion of a Burch–Schneider ring [44].

### 2.4. Comparison among Models

The two models present some common points as well as some major differences. In this respect, the most important common aspect is related to the stress distribution on the internal part of the pelvis, corresponding to the sacroiliac joint, and on the pubic ramus. Here, the stress values were in the range of 20–40 MPa and the same maximum values were recorded (50–60 MPa). This general distribution is also comparable with the one found by Dalstra et al. [52,53] for a similar case.

On the other hand, major differences were recorded on the retroacetabular region. In the pathological model treated with a Burch–Schneider ring, a significant part of the force was adsorbed by the inserted plate; indeed, the value of the stress was reduced to 3 MPa. This had an effect on the surrounding bone, which has to withstand stresses that are higher than the physiological ones. As a matter of fact, if the plate were removed, the entire bone structure would fail due to its lower stiffness. Furthermore, as, in practice, the thickness of the bone section behind the metal back would be very small, it would hardly be possible to apply the load in the pathological patient without any type of auxiliary structure. The model treated with SmartBone^®^, on the other hand, showed that the bone substitute was not characterized by high levels of stress (3 MPa), and the stresses on the surrounding bones were in line with the conventional ones observed in physiological cases (3–6 MPa). Nevertheless, the high level of displacement (1–1.5 mm) of the insert indicates that, in order to achieve effective structure stability, the use of a plate would be more necessary than an option. Indeed, it would properly prevent the consequential push on the retroacetabular residual bone, which, without any fixation system, would probably not be able to support the applied loads and eventually collapse.

## 3. Discussion

Significant values of stress are present in the hip–pelvis complex. Therefore, the treatment of a pathological patient with a relevant defect on the acetabular zone is not so simple to study. The surgical complexity of these cases is further worsened by risks of osteopenic femur head syndrome and overall bone grafting reabsorption in the postop timeframe. These cases, in general, are treated with a relatively invasive solution. On the other hand, the potentialities of SmartBone^®^ as a valid alternative to the common surgical practice have been highlighted and confirmed both by the experimental tests and computational simulation. Indeed, the stress distribution shows that over the whole volume of the graft, the stress is always below the maximum withstood by the material. Moreover, the values in the surrounding bones are comparable to the physiological ones. These findings corroborate the possibility of performing THA surgeries without the insertion of an invasive metal support, such as a Burch–Schneider ring. In addition, they can be nicely combined with the cell adhesion and osteointegration characteristics of the material and can be remodeled, in other districts, in less than a couple of years, enabling better bone regeneration and opening a path to a new approach to THA primary or revisional surgeries. Of course, the specific nature of the bone and its potential previous defects in the acetabular region should be considered for each patient (also considering age, gender, etc.). Reasonably, the application of a thinner metal (e.g., titanium) plate, as a fixation system, between the patient’s bone and the graft has to be considered for the worst cases.

## 4. Materials and Methods

### 4.1. Mechanical Characterization

#### 4.1.1. Sample Preparation

*Cylindrical samples.* Each sample had a nominal diameter of 10 mm and a height of 25 mm and was connected to two stainless-steel endcaps using an acrylic resin (Technovit^®^ 6091—Kulzer Technique, Wehrheim, Germany). The specimen–endcap complex was then gripped between the jaws of the testing machine. Cylindrical specimens were used both for compression and torsion tests.

*Rod samples.* Each sample had a length of 60 mm and a square cross section, the side of which was 7 mm. The sample was put between the rollers of a four-point bending bench, which was connected to the testing machine.

#### 4.1.2. Testing Procedures

Tests were performed on an MTS 858 Bionix servohydraulic testing machine (S/N 1014952, MTS, Minneapolis, MN, USA). The MTS testing machine was equipped by an axial/torsional hydraulic actuator that had a 25 kN axial capacity and a 250 Nm torsional capacity, a ±100 mm range LVDT displacement transducer, and a ±140° range ADT angular transducer mounted on the actuator. The load applied to the test sample was measured by an MTS axial/torsional load cell. (model 662.20D-05, S/N 1007099; ±25 kN maximum axial load and ±250 Nm maximum torsional load). The machine was driven by a Test Star 790.01 digital controller (MTS, Minneapolis, MN, USA).

*Compression tests.* In accordance with Keaveny et al. [4], 20 cylindrical samples were used for this type of test. Tests were run under displacement control at a velocity of 0.5 mm/s until failure of the specimen. During the test, the data of force and displacement were recorded at a 10 Hz frequency. Six preconditioning cycles ranging from 50 to 500 N were performed before the final step to rupture. From the data of force and displacement recorded during the test, stress and strain were calculated as follows:

(1) $$\sigma =\frac{F}{A}$$

where F is the force applied by the actuator and A is the initial circular cross section area. Further,

(2) $$\varepsilon =\frac{\Delta l}{l_{0}}$$

where Δl is the vertical displacement recorded during the test and l_0_ is the initial length of the specimen. Finally, the elastic modulus E was calculated as a linear regression of the stress–strain curve in the elastic region.

*Bending test.* Twelve rod specimens were tested using the four-point bending setup used by Draper et al. [5]. Moreover, for the bending tests, six preconditioning cycles between 5 and 50 N were applied before the final ramp to rupture. The test speed was also in this case equal to 0.5 mm/s.

From the data of force and displacement recorded during the test, stress and strain were calculated as follows:

(3) $$\sigma =\frac{F\cdot \left(L-C\right)/2}{J_{F}}\cdot y$$

where F is the applied force, L is the distance between the lower rollers of the bench, C is the distance between the upper rollers of the bench, J is the bending moment of inertia of the specimen, and y is the distance of the section where stress is calculated from the neutral axis. Further,

(4) $$\varepsilon =\frac{6\cdot \Delta l\cdot y}{\left(L-C\right)\cdot \left(L+2C\right)}$$

where Δl is the vertical displacement recorded during the test and all the other parameters have already been defined. Finally, the elastic modulus E was again calculated as a linear regression of the stress–strain curve in the elastic region.

*Torsional test.* The set of samples was a group of 10 SmartBone^®^ samples (lot TT). Tests were run under angular displacement control at a speed of 0.1°/s according to [6]. All the samples were preconditioned with six preconditioning cycles between a minimum torque value of −600 Nmm and a maximum one of 600 Nmm before the ramp to rupture.

From the data of torque and angle recorded during the test, tangential stress and angular strain were calculated as follows:

(5) $$\tau =\frac{M_{t}}{J_{T}}$$

where M_t_ is the torque measured by the load cell and J_T_ is the torsional moment of inertia of the specimen. Further,

(6) $$\gamma =\frac{\alpha \cdot r}{l_{0}}$$

where α is the angle measured during the test, r is the radius of the specimen, and l_0_ is the initial length of the specimen. Finally, the tangential modulus *G* was calculated as a linear regression of the tangential-stress–strain curve in the elastic region.

### 4.2. ESEM Analysis

To evaluate the SmartBone^®^ structure, an EVO^®^50 ZEISS scanning electron microscope (Zeiss AG, Oberkochen, Germany was employed. It was characterized by a very large specimen chamber (356 (Ø) × 255 mm (h)). The analysis was performed by imposing the extended pressure vacuum mode, with a pressure value of 40 Pa. Images were taken point by point. After the signal detection, the software SmartSEM (Zeiss AG, Oberkochen, Germany)). was used to process data.

### 4.3. Models

#### 4.3.1. Development of 3D Physiological Pelvis Model

In order to validate the models created—one treated with a Burch–Schneider ring and the other one with SmartBone^®^—a physiological model from the literature was adapted (Dalstra et al. [52,53]). CT scan images were used to build the models. Regarding the healthy patient, the dataset used to validate the model was provided by University of Iowa Health Care [54] and it referred to a healthy human female (age 60) without any particular complication on the pelvic bone.

Virtual models of the pelvis were reconstructed using the Mimics Innovation Suite Research 20.0 (Materialise, Leuven, Belgium) (MIS). Grayscale DICOM images was used to recognize and discretize the bone. A specific thresholding level was automatically set by Mimics and it referred to both the trabecular and cortical bones. In this case, threshold levels were set at 226 HU as a minimum and at 1771 HU for the higher one. These values cannot be considered absolute due to many factors that influence HU level, such as the specific scanner being used, the energy settings, and the object being scanned. Furthermore, other patient-specific parameters influence this level, such as the age of patient and the density of the bone [55].

The quality of the model was improved in MIS using different functions (built-in Mimics software (Materialise, Leuven, Belgium) functionalities). In particular, after the acquisition of the model, the surface of the model was treated in order to improve the mesh quality. A first reconstruction step was finished filling the internal volume. This aspect was important to understand the behavior of the model in its internal part.

The mesh of the model was refined always using the tools present in MIS with elements characterized by a length of 2–3 mm, since the quality of the automatic mesh was not good to perform the functional analysis required to simulate a load condition.

Finally, a volumetric mesh was built using the same size mesh already adopted for the superficial elements. The element type chosen to discretize the pelvis model was C3D4 and consisted of a tetrahedral linear element.

##### Assigned Material Properties

The meshed model obtained in MIS was not characterized by material properties. This step was scheduled by again importing the model in Mimics and using the grayscale criteria. In detail, a specific number of intervals was chosen to characterize different material density (ρ) and Young’s modulus (E) values. These values were obtained through the following equations [56]:

(7) ρ=1.067×HU+131

(8) $$E=0.004\times \rho^{2.01}.$$

The Poisson’s ratio was fixed to a value of 0.3, in line with the literature [57]. The final volume mesh model with the assigned material properties was then exported as an input file to Abaqus CAE for the final step of finite element analysis (FEA). The material properties used in the test simulation for SmartBone^®^ were the ones determined via mechanical characterization.

##### Load Applications, Constraint Definitions, and Boundary Conditions

The next step in performing an exhaustive FEA was to apply all forces referring to the hemipelvis–femur complex. They included both muscle and reaction forces. As shown by Dalstra et al. [53], the value of the forces varies during the walking cycle. In particular, the most significant value of hip joint force is related to the beginning of the single support phase. Here, the reported value was 2148 N.

The hip reaction force was applied in the center of the acetabular cup and then distributed on the whole acetabulum using a reference point (RP) previously created. The force application angle was set to 50° in the *x*–*z*-plane to simulate the inclination angle of the femoral head [58].

Then, another revolution of 18° clockwise was done, twisted around the *x*-axis to simulate the flexion in phase two of the walking cycle.

All forces applied were distributed on the complex by the RP, establishing a specific contact between the RP and the surface considered. The type of constraint adopted in this case was the “coupling” one.

Regarding muscle forces, a medium value of insertion points on the hemipelvis was found by Dostal et al. [59]. Furthermore, they provided the coordinates of distal points of the same muscles. In this way, the principal direction of the fibers was implicitly given.

Then, a set of straight lines was found, one for each couple of points—distal and proximal. Each straight line generated was then used to identify a plane that was normal to the line. In particular, the distal point of each couple was used as a common point between the plane and a straight line. Another point was then created on the plane previously generated. This was necessary to obtain a new coordinate system, in which the *z*-axis represented the direction of the application of the load. This procedure was realized for each muscle. All forces, except for the hip one, were related to muscles inserted into the pelvis.

**Figure 7:**
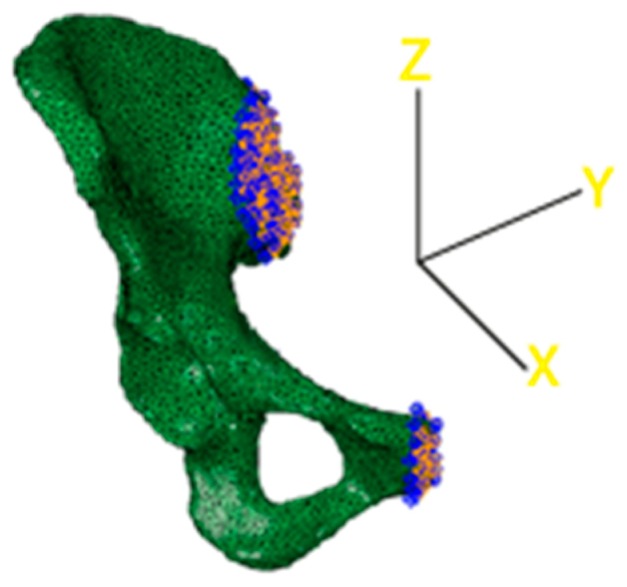
3D reconstruction of the acetabulum model. Constraints are highlighted in blue.

The final step consisted of the choice of the boundary conditions that blocked different degrees of freedom of the model. In this case, two different sets of nodes were considered to encastre the model. The first one was related to the interface between the upper part of the iliac crest and the lateral surface of the sacrum, as visible in Figure 7. The second one was positioned corresponding to the end of the pubic ramus to avoid excessive displacements of the lower part of the pelvis. Nodes to bind were chosen only on the external surfaces, facing the lateral section of the sacrum and the lateral section of the pubic ramus of the complementary hemipelvis.

Although the physiological behavior allowed small displacements both in the upper part of the iliac crest and the lateral part of the pubic ramus, the hemipelvis was fixed with two encastre boundary conditions. At this point, the model was ready for the computational simulation.

##### Behavior of the Physiological Model

**Figure 8:**
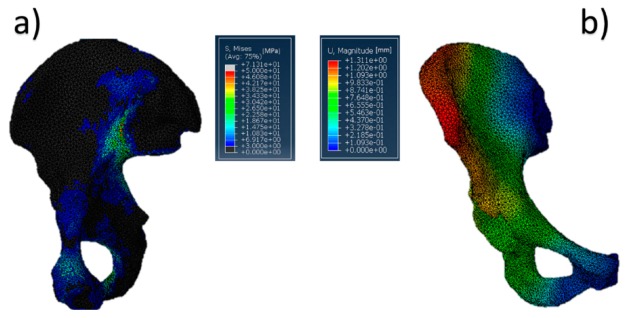
Acetabulum physiological model: (**a**) von Mises stress distribution (data are expressed in megapascals) and (**b**) displacement distribution (data are expressed in millimeters).

The behavior of the developed physiological model is shown in Figure 8, which shows phase two of the cycle, because in that phase, the value of the hip force is the largest one [53].

The most significant values of stress (Figure 8a) were both close to the pubic symphysis and the ischium, whereas the most relevant displacement was concentrated in the upper part of the iliac crest. Also relevant is the absence of substantial stresses into the acetabular cup. This aspect cannot be considered completely true due to the fact that Abaqus CAE does not identify the value of contact pressure in homogeneous or semihomogeneous materials. Concerning the stress distribution along the pelvis, the high values found can be attributed both to all forces present in the model and the boundary conditions chosen. However, the most significant contribution was provided by the hip reaction force. In fact, the intensity of this load was at least twice that of the other muscle loads. Furthermore, all muscle forces try to equilibrate the pelvis. The maximum value reached in the model was around 71 MPa, but all of the highest values (over 45 MPa) were concentrated in the iliac sacral junction. On the other hand, these values are punctual and do not represent a wide stress distribution in a specific zone. Finally, the choice to encastre the model was also functional to avoid excessive displacement of the acetabular region. In this way, acetabular displacements were limited to 0.5–0.6 mm, and this can be considered acceptable for the purpose of this work.

#### 4.3.2. Development of the 3D Pelvis Pathological Models

For the development of the pathological models, CT scan images were obtained by San Martino Hospital of Genova. The dataset referred to a male patient (age 80) already treated with a first prosthesis implant which needed a revision surgery due to the critical conditions of the hemipelvis complex. The same technique used to obtain and simulate the physiological model was again adopted in the pathological cases. The only significant difference between physiological and pathological models was the presence of an additional structure anchored to the pelvis during the revision surgery. The conventional technique allows the insertion of a metallic graft (a Burch–Schneider ring), which is fixed to the pelvis with seven different screws. In the second simulation, the same dataset was used. The inserted component was a SmartBone^®^ graft, the dimensions of which were obtained by studying pre- and postrevision surgery CT images and performing a Boolean subtraction on them on MIS. This operation allowed us to obtain a rational value of the diameter and thickness of the graft that was inserted in the final model.

Due to the presence of this component, it was also important to define another constraint to simulate the contact between the component and the model. It was decided to apply the contact as a “Tie” constraint to fix the two parts together.

Finally, the load conditions and boundaries used to simulate the pathological pelvis were the same as those used in the physiological case.

## Figures and Tables

**Table 1:** Mechanical properties for compression, bending, and torsion of SmartBone^®^.

Test	Max Stress (MPa)	Max Strain (-)	Elastic Modulus (GPa)
Compression	25.8 + 7.9	0.024 + 0.005	1.2456 + 0.2259
Bending	23.8 + 4.2	0.0765 + 0.009	0.3406 + 0.063
Torsion	25.5 + 4.4	0.058 + 0.009	0.4906 + 0.1037

## Supplementary Materials

The following are available online at https://www.mdpi.com/2079-4983/10/4/53/s1, Table S1: Full report of compression tests. Table S2: Full report of bending tests. Table S3: Full report of torsional tests. Figure S1. Stress–strain data for compression tests. Figure S2. Stress–strain data for bending test. Figure S3. Stress–strain data for torsional tests.
