# Supplementary material for: Simulated Performance of a Xenohybrid Bone Graft (SmartBone®) in the Treatment of Acetabular Prosthetic Reconstruction
Source: J Funct Biomater. 2019 Nov 22;10(4):53. doi: 10.3390/jfb10040053 (PMC6963854; doi:10.3390/jfb10040053)
Supplement: Supplementary file 1 [file jfb-10-00053-s001.pdf]

Journal of Functional Biomaterials

MDPI

Supporting Information

Simulated Performance of a Xenohybrid Bone Graft (SmartBone®) in the Treatment of Acetabular Prosthetic Reconstruction

Carlo Francesco Grottoli [1], Alberto Cingolani [1], Fabio Zambon [2], Riccardo Ferracini [3,4], Tomaso Villa [2] and Giuseppe Perale [1,5,*]

1 Industrie Biomediche Insubri SA, 6805 Mezzovico-Vira, Switzerland; carlo.grottoli@ibi-sa.com (C.F.G.); alberto.cingolani@ibi-sa.com (A.C.)
2 Politecnico di Milano, Laboratory of Biological Structure Mechanics, Department of Chemistry, Materials and Chemical Engineering "G. Natta", 20133 Milan, Italy; fabio.zambon@mail.polimi.it (F.Z.); tomaso.villa@polimi.it (T.V.)
3 Department of Surgical Sciences and Integrated Diagnostics, University of Genova, Largo R. Benzi 10, 16132 Genova, Italy; riccardoferraciniweb@gmail.com
4 IRCCS Ospedale Policlinico San Martino, Largo R. Benzi 10, 16132 Genova, Italy
5 Ludwig Boltzmann Institute for Experimental and Clinical Traumatology, Donaueschingenstrasse 13, 1200 Vienna, Austria
* Correspondence: giuseppe@ibi-sa.com; Tel.: +41-91-930-6640

Table S1. Compression tests results. The maximum value of force (F_{max}), stress (σ_{max}), strain (ε_{max}) and the value of Young's Modulus (E) are reported for each sample. All values are referred to elastic region of stress-strain curve. The Young's Modulus is calculated as linear regression of the punctual values. Dimensions for cylinders are expressed as diameter (d) times height (h).

Lot-Sample	F_{max} [N]	σ_{max} [MPa]	ε_{max}	E [GPa]	dxh [mmxmm]
271-1	1833.5	24.91	0.026	0.9501	9.81 × 24.64
271-2	1597.9	21.75	0.025	0.9223	9.77 × 24.67
271-3	1530.3	20.90	0.020	0.9216	9.69 × 24.3
271-4	2218.3	30.07	0.023	1.2548	9.8324.54
**Medium Value 271**	**1795**	**24.41**	**0.024**	**1.0122**	–
**Standard Dev. 271**	**310.7**	**4.15**	**0.003**	**0.1623**	–
272-1	2691.9	36.27	0.029	1.2561	9.77 × 24.49
272-2	1125.8	15.26	0.015	1.1858	9.84 × 24.62
272-3	3172.3	42.48	0.028	1.7306	9.8 × 24.72
272-4	1907.3	25.58	0.020	1.4041	9.82 × 24.45
**Medium Value 272**	**2224.3**	**29.90**	**0.023**	**1.3942**	–
**Standard Dev. 272**	**899.0**	**12.00**	**0.007**	**0.2421**	–
273-1	1030.3	13.91	0.017	0.9525	9.86 × 24.67
273-2	1692.4	23.04	0.023	1.1652	9.81 × 24.7
273-3	1941.4	26.43	0.028	1.3646	9.76 × 24.67
273-4	1913.0	25.94	0.024	1.0995	9.77 × 24.66
**Medium Value 273**	**1644.3**	**22.33**	**0.023**	**1.1455**	–
**Standard Dev. 273**	**424.2**	**5.81**	**0.00455**	**0.1710**	–
274-1	841.7	11.33	0.014	0.8036	9.83 × 24.7
274-2	710.0	9.74	0.017	0.5756	9.84 × 24.68
274-3	798.9	10.96	0.018	0.7201	9.69 × 24.72
274-4	927.0	12.36	0.013	1.0712	9.8 × 24.45

**Medium Value 274**	**819.4**	**11.10**	**0.016**	**0.7889**	–
**Standard Dev. 274**	**90.3**	**1.08**	**0.002**	**0.2549**	–
275-1	1113.9	14.97	0.014	1.1738	9.82 × 24.71
275-2	1689.9	22.83	0.022	1.1222	9.84 × 24.48
275-3	2467.2	32.46	0.021	1.6792	9.87 × 24.67
275-4	2701.2	35.76	0.021	1.7470	9.77 × 24.63
**Medium Value 275**	**1993.1**	**26.51**	**0.020**	**1.4306**	–
**Standard Dev. 275**	**728.3**	**9.45**	**0.004**	**0.3281**	–

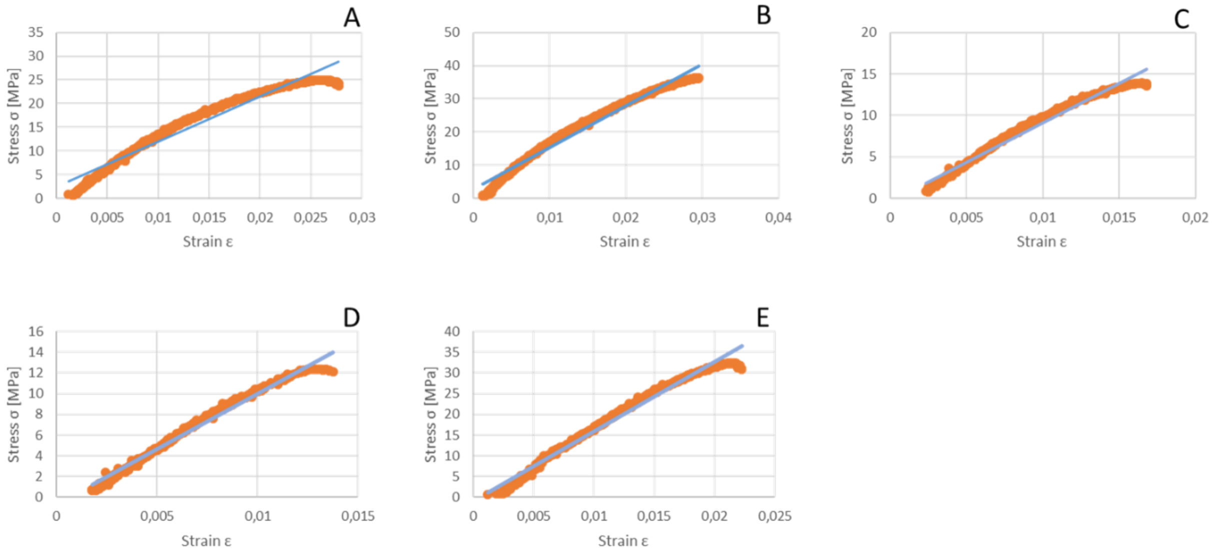

Figure S1. Exemplificative stress-strain data obtained during compression test. Specifically, (**A**) refers to dataset 271, (**B**) to 272, (**C**) to 273, (**D**) to 274 and (**E**) to 275.

Table S2. Bending test results. The maximum value of force (F_{max}), stress (σ_{max}), strain (ε_{max}) and the value of Young's Modulus (E) are reported for each sample. All values are referred to elastic region of stress-strain curve. The Young's Modulus is calculated as linear regression of the punctual values. Dimensions of the bars (parallelepiped shape) are expressed as first length (a) times second length (b) times third length (c).

**Lot-Sample**	**F_{max} [N]**	**σ_{max} [MPa]**	**ε_{max}**	**E [GPa]**	**axbxc [mmxmmxmm]**
276-1	116.0	27.88	0.074	0.4156	7.14 × 7.14 × 58.45
276-2*rott	71.6	17.36	0.067	0.2320	7.11 × 7.11 × 58.32
276-3	118.3	28.92	0.078	0.3687	7.10 × 7.10 × 58.48
276-4	105.6	25.01	0.063	0.4112	7.17 × 7.17 × 58.38
276-5	96.5	22.63	0.065	0.3741	7.20 × 7.20 × 58.34
276-6	95.3	22.45	0.076	0.3245	7.18 × 7.18 × 58.32
**Medium Value 276**	**100.6**	**24.04**	**0.071**	**0.3543**	–
**Standard Dev. 276**	**17.1**	**4.21**	**0.006**	**0.0685**	–
277-1	120.3	28.18	0.090	0.3439	7.21 × 7.21 × 58.62
277-2	85.3	19.99	0.064	0.3739	7.21 × 7.21 × 58.72
277-3	122.8	28.78	0.075	0.3992	7.21 × 7.21 × 58.71
277-4	101.3	23.63	0.087	0.2978	7.22 × 7.22 × 58.64
277-5	87.2	20.73	0.075	0.3065	7.18 × 7.18 × 58.70
277-6	83.4	19.66	0.098	0.2400	7.20 × 7.20 × 58.69
**Medium Value 277**	**100.0**	**23.50**	**0.082**	**0.3269**	–
**Standard Dev. 277**	**17.8**	**4.11**	**0.012**	**0.0575**	–

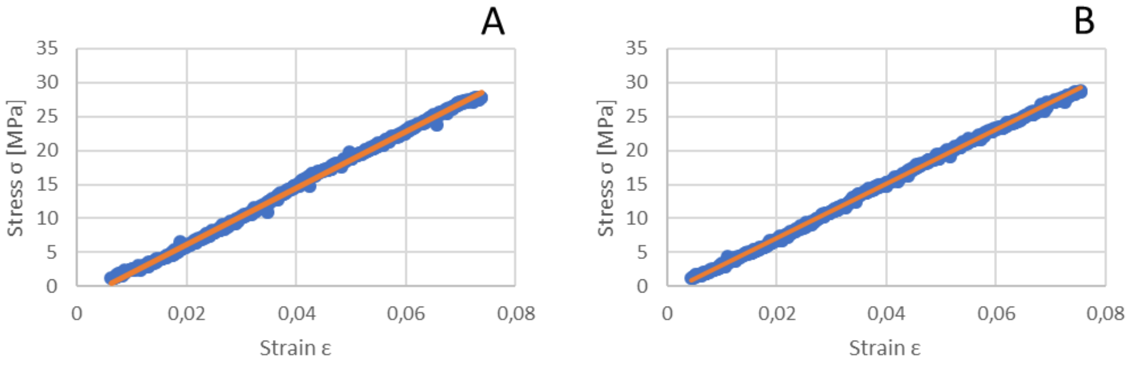

Figure S2. Exemplificative stress-strain data obtained during bending test. Specifically, (**A**) refers to dataset 276 and (**B**) to 277.

Table S3. Torsional test results. The maximum value of torque (Tmax), stress (τ_{max}), strain (γ_{max}) and the value of tangential elastic Modulus (G) are reported for each sample. All values are referred to elastic region of stress-strain curve. The G Modulus is calculated as linear regression of the punctual values. Dimensions for cylinders are expressed as diameter (d) times height (h).

Lot-Sample	$Torque_{max}$ [Nmm]	τ_{max} [MPa]	γ_{max}	G [GPa]	dxh [mmxmm]
TT-1	997.6	17.60	0.055	0.3555	9.89 × 24.64
TT-2	1042.4	18.88	0.062	0.3472	9.69 × 24.68
TT-3	1522.8	25.47	0.058	0.5297	9.83 × 24.67
TT-4	1696.7	28.63	0.046	0.6179	9.77 × 24.59
TT-5	1418.1	23.55	0.058	0.4456	9.8 × 24.27
TT-6	1553.5	26.45	0.082	0.4341	9.81 × 24.67
TT-7	1675.4	27.60	0.063	0.4782	9.87 × 24.49
TT-8	1461.2	25.58	0.055	0.4490	9.81 × 24.72
TT-9	1629.6	27.58	0.053	0.5610	9.75 × 24.66
TT-10	2057.0	33.48	0.051	0.6880	9.85 × 24.39
**Medium Value TT**	**1505.4**	**25.48**	**0.058**	**0.4906**	–
**Standard Dev. TT**	**294.9**	**4.40**	**0.009**	**0.1037**	–

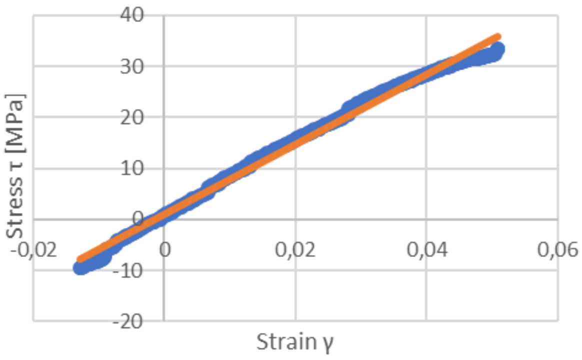

Figure S3. Exemplificative stress-strain data obtained during torsion test.

© 2019 by the authors. Submitted for possible open access publication under the terms and conditions of the Creative Commons Attribution (CC BY) license (http://creativecommons.org/licenses/by/4.0/).
